# High-pressure continuous culturing: life at the extreme

**DOI:** 10.1128/aem.02010-24

**Published:** 2025-01-22

**Authors:** Dionysis I. Foustoukos, Jennifer L. Houghton

**Affiliations:** 1 Earth and Planets Laboratory, Carnegie Institution for Science 613012 , Washington, DC, USA; 2 Department of Earth, Environmental and Planetary Sciences, Washington University in St. Louis 7548 , St. Louis, Missouri, USA; University of Delaware, Lewes, Delaware, USA

**Keywords:** high-pressure, continuous culturing, chemostat, piezophiles, extremophiles, chemolithoautotrophs, heterotrophs, hydrothermal vents, pressure adaptation

## Abstract

Microorganisms adapted to high hydrostatic pressures at depth in the oceans and within the subsurface of Earth’s crust represent a phylogenetically diverse community thriving under extreme pressure, temperature, and nutrient availability conditions. To better understand the microbial function, physiological responses, and metabolic strategies at *in-situ* conditions requires high-pressure (HP) continuous culturing techniques that, although commonly used in bioengineering and biotechnology applications, remain relatively rare in the study of the Earth’s microbiomes. Here, we focus on recent developments in the design of HP chemostats, with particular emphasis on adaptations for delivery and sampling of dissolved gases. We present protocols for sterilization, inoculation, agitation, and sampling strategies that minimize cell lysis, applicable to a wide range of chemostat designs.

## INTRODUCTION

Technological advances in high-pressure (HP) microbial culturing provide the means to understand microbial physiology, function, and adaptation mechanisms under physicochemical conditions considered extreme relative to ambient pressures and temperatures. The application of HP culture studies extends from the extreme habitats of deep oceans on Earth and icy satellites to the biotechnology of fossil fuel research to developing sterilization protocols for the food industry. For example, the majority of Earth’s biosphere lives at the ocean’s great depths and within the subsurface of the oceanic crust (1, 2). This deep biosphere thrives under conditions of high hydrostatic pressures (>10 MPa) by utilizing metabolic strategies that permit adaptation to a wide range of pressure, temperature, and nutrient availability (3–6). The HP adapted organisms (piezophiles) exhibit a phylogenetic diversity that extends to all three domains of life (7). Such highly diverse microbiomes exist near deep-sea hydrothermal vents, where organisms have developed adaptations not only to pressure (>20 MPa) but also to sharp gradients in temperature (4 to >100°C) and redox (8–10). Deep-sea hydrothermal systems on Earth are considered proxies to investigate microorganisms' physiological and metabolic adaptations at conditions that may resemble those found on icy planetary bodies such as Enceladus and Europa (11–14).

Microbial culturing under high pressure also holds significant importance in industrial biotechnology for the development of biofuel/bioremediation procedures (15–17) and to facilitate sterilization of food resources at ambient temperature (18–20). Food pasteurization (i.e., pascalization) associated with microbial inactivation under HP conditions increases the shelf life without altering the nutritional value and quality of processed food as expected during traditional pasteurization processes (e.g., references 19, 21). In bioengineering, HP culturing of microorganisms associated with fossil-fuel subsurface reservoirs, if implemented, has the potential to provide important insights into our understanding of microbial-mediated biodegradation processes (15, 17, 22) and diesel-like fuel biosynthesis (23, 24). Environmental studies on the effect of fossil fuel contamination in marine environments have also expanded to include HP culturing of microbial communities retrieved from the deep oceanic seafloor (25).

**Fig 1:**
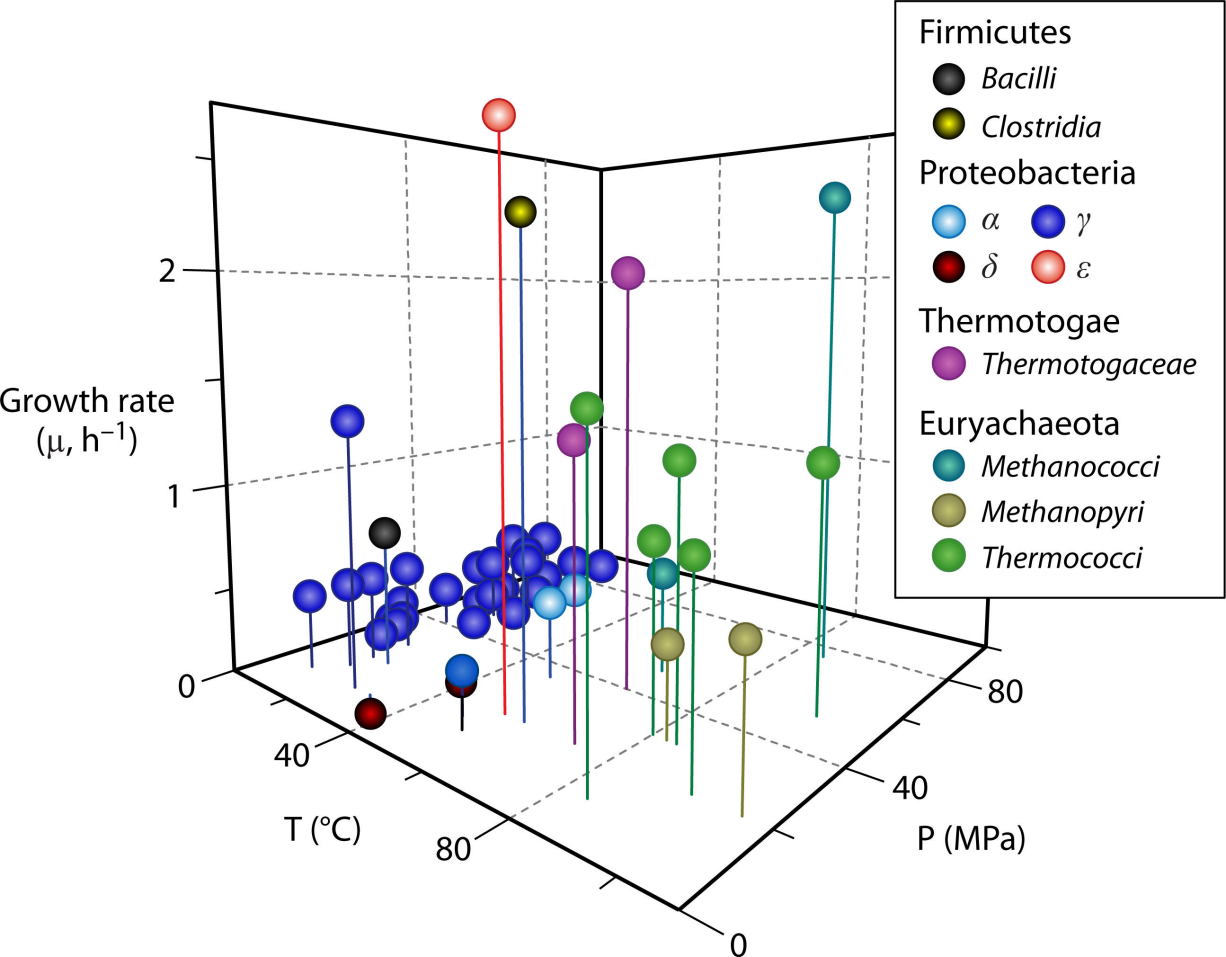
Growth rates of piezophilic microorganisms cultured at different pressure and temperature conditions. Data are from Jebbar et al. (7) and Smedile et al. (36).

Considering the contribution of the piezophilic microbial communities to the Earth’s microbiomes and recent biotechnological advances, it is striking how little we know about the function and physiological responses of piezophiles to extreme physical conditions mainly driven by the paucity of HP culture studies (7) (Fig. 1). From the limited HP cultures available, cellular membrane and related membrane proteins, such as those involved in the bacterial secretion system, are probably the most pressure-sensitive biological structures (1, 4, 26). Piezophilic microorganisms have been shown to alter the viscosity of their membrane lipids to maintain the necessary fluidity and permeability so that substrates and waste can quickly diffuse across cellular membranes (27–30). Energy production and central metabolism-related genes also appear to be overexpressed under high hydrostatic pressures, along with the upregulation of genes involved in amino acid/carbohydrate synthesis and respiratory functions (31–35). Given the dearth of HP cultivation experiments, integrating culture-based studies of the physiological and metabolic functions of piezophilic microorganisms with genomics is critical for revealing the microbial adaptation strategies in these extreme environments. Here, we present recent developments, protocols, and future directions associated with culturing microorganisms at high pressures and temperatures.

## HP CULTURING TECHNIQUES

### Batch culturing

The majority of methods developed for HP microbial culturing involve batch culturing that employs stainless steel high-pressure reactors and uses H_2_O as the pressure-transmitting medium. “HP,” therefore, corresponds to “high hydrostatic pressures.” Batch culturing approaches are relatively easy to use and allow pressures exceeding 100 MPa. Early experimental studies paved the way for the technological advances required to culture piezo-philic/tolerant microorganisms collected from abyssal oceanic environments under challenging culturing conditions (e.g., anaerobic, H_2_/N_2_/CO_2_ headspace) (37–43). In the protocols developed, the actual cultures are enclosed in syringes, plastic bulbs/bags, or Hungate tubes placed inside the pressure vessels (44–51). In batch cultures, subsampling occurs by terminating the experiment for direct recovery of the cultured biomass.

Most HP batch studies address the physiology, function, or adaptation mechanisms of monocultures. In batch cultures, experiments are terminated either at the exponential or the stationary growth phase; phase conditions that have been predetermined from cultures at ambient pressure. Batch culturing conducted at the optimum physicochemical conditions (T, P, and nutrient availability) for growth permits the upper limits of biomass formation to be assessed, yielding estimations of maximum growth rates and biomass levels sufficient to perform molecular analyses (e.g., proteomics and transcriptomics) (32, 34, 35, 46, 52–60). The employment of several culture-containing reaction cells (e.g., syringes, Hungate tubes, and microchannels) in a single pressure vessel permits experimental replicates to be conducted simultaneously. The major limitations of this batch culturing approach are the small volume (<20 mL) of the reaction cells and the efficiency of monitoring the different stages of microbial growth by time-series subsampling. Furthermore, the effect of nutrient depletion on the growth of microorganisms is challenging to assess, considering that substrate levels are not kept constant at the different growth phases.

The development of large volume (>50 mL) batch reactors, often constructed using chemically inert materials, that can facilitate subsampling along all stages of growth at pressure (e.g., references 42, 61, 62) has proven to be effective for the culturing of mixed communities and for monitoring the evolution of key chemical (headspace gases and soluble substrates) and molecular (e.g., gene expression) aspects of the HP cultures. In the case of pressure vessels with chemically inert wall surfaces (PEEK, Au, Ni, Ti alloys, and sapphire), designs can allow for sample retrieval at *in situ* pressure with subsequent culturing conducted at HP directly in the batch reactor (e.g., references 41, 42, 61–72). In these designs, the direct introduction of growth medium into the bioreactor while sampling allows for time-series samples to be collected during the experiment with minimal effects on the growth of the microorganisms inside the bioreactor. Here, the volatile composition (e.g., H_2_, CO_2_, and CH_4_) of the growth medium can be determined at conditions by withdrawing the samples into a gas-tight syringe.

In these bioreactors, the effect of pressure, temperature, and nutrient availability/speciation on the functional and physiological diversity of anaerobic chemolithoautotrophic bacteria can be explored at *in-situ* conditions. For example, McNichol et al. (67, 73) conducted a series of batch experiments at 25 MPa and 25–50°C with mixed natural communities collected from a deep-sea hydrothermal vent on the Pacific Ocean (9°N East Pacific Rise). The batch reactor used was an isobaric gas-tight vent fluid sampler (150 mL) that permits the culturing of vent microorganisms under the *in-situ* conditions of their subsurface oceanic habitat (2,500 m water depth). Recently, Pérez-Rodríguez et al. (74), Zhang et al. (66), Frerichs et al. (63) performed HP batch culturing by employing large volume flexible Au/Ti reaction cells (50 mL) placed inside a pressure vessel. A titanium exit tube and sampling valve attached to the reaction cell allow multiple internally filtered fluid samples to be obtained during an ongoing experiment (75). In these designs, the chemical composition of the growth medium can also be modified by the *in-situ* injection of gas-enriched aqueous solutions directly into the Au/Ti cell or the isobaric gas-tight sampler. Most importantly, pressure conditions are maintained constant during sampling because the volume of the sample removed is replaced by an equivalent volume of pressure transmitting medium (H_2_O) surrounding the Au/Ti cell or the incubation chamber of the isobaric gas-tight sampler.

Culturing at extremely high pressures (>1,000 MPa) has been conducted by using diamond anvil cells of minuscule volumes (<<1 μL) (76–78). In these experiments, optical microscopy and *in-situ* spectroscopy (Raman, μXANES) provide the means to monitor the growth (or the lack of) by direct cell counting or by assessing the relative abundances of growth substrates (e.g., references 79–81).

### Continuous culturing

The development and utilization of HP continuous culturing techniques are less common than the batch culturing approaches, probably because of the higher complexity of the experimental design. Some of the earliest technological advances in HP continuous culturing are reported by Miller et al. (82). The pioneering work of Jannasch et al. (83) and Wirsen and Molyneaux (84) showed the critical attributes of continuous culturing to constrain the physiological functions and adaptation mechanisms of piezophilic organisms not only to fluctuating pressure conditions but also to the availability of growth substrate. In short, these experimental setups allowed for the delivery of growth medium enriched in dissolved gases and soluble substrates while permitting periodic sampling of the incubated organisms. According to the fundamental concepts of continuous culturing (e.g., reference 85), adjusting flow rates of fluid (influent) delivery controls microorganisms' growth rate inside the bioreactor. In this way, cultures can be maintained under a steady-state cell density to collect biomass samples large enough for quantitative molecular analysis. Essential elements of the design are: (i) an agitation mechanism to facilitate the homogenization of the medium solution and cultures, and (ii) a backpressure-regulating valve that maintains the system under constant pressure and medium delivery rates. In general, these are large volume bioreactors (>100 mL) that can sustain pressures of up to 71 MPa (e.g., references 83, 86) for the culture of hyperthermophilic microorganisms (*T* > 80°C). HP bioreactors of much smaller volumes (<10 mL) have been developed for *in-situ* optical and spectrophotometric observations of living cells (87–92). The continuous culture approach provides a unique opportunity to study microbial activity and functions by adjusting pressures and substrate compositions dynamically. This represents a critical difference that sets this experimental strategy apart from mainstream batch mode approaches. A detailed review of several techniques applied to marine biotechnology (batch and continuous culturing) is presented in Roy (93).

To our knowledge, there have been only nine HP continuous culturing systems (chemostats) that can reach pressures ≥10 MPa (62, 83, 86, 94–99). The applications of these designs have mainly been limited to culturing mixed natural communities sampled from deep marine environments (94, 95, 100). For example, Houghton et al. (94) cultured the anaerobic, nitrate- and sulfate-reducing chemolithoautotrophic bacteria attached to sulfide structures from hydrothermal vents at 9°N EPR at high hydrostatic pressure (25 MPa) and hyperthermophilic temperatures (70–90°C). By adopting these earlier approaches, recent developments have permitted the continuous culturing of pure and mixed chemolithoautotrophic microbial communities at 25–120°C and pressures as high as 69 MPa (36, 86, 100, 101). Here, we discuss recent developments, protocols, and future directions associated with HP continuous culturing approaches.

## CULTURING PROTOCOLS

### Designing a HP chemostat

**Fig 2:**
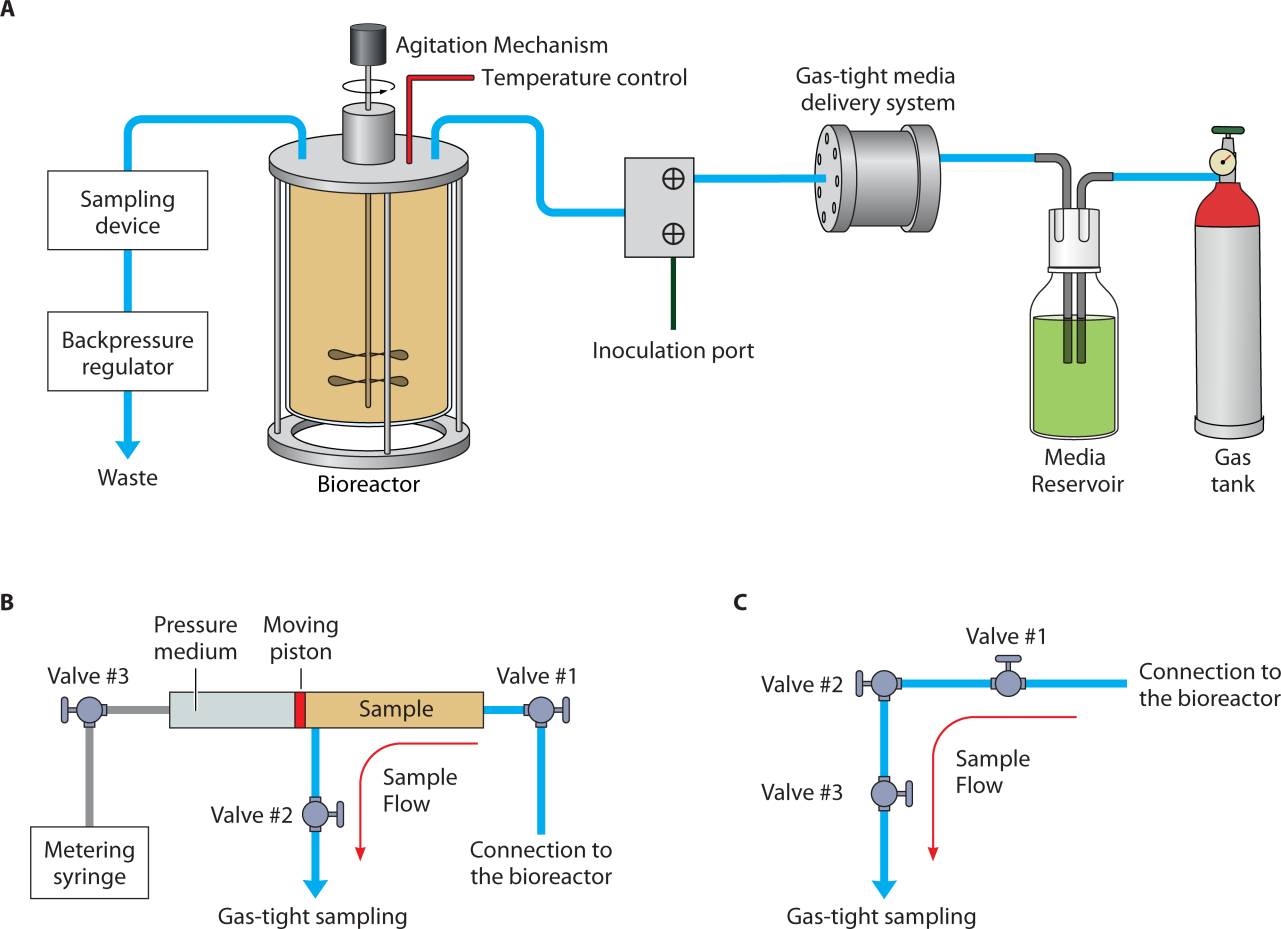
Culturing and sampling microorganisms at HP conditions. (**A**) Schematic depiction outlining the critical components of a HP continuous culturing design (modified from reference 86). Sampling devices that allow subsampling from HP cultures are: (**B**) the sampling arrangement of Taylor and Jannasch (39) that uses a moving piston to separate the sample collected from a pressure transmitting medium and to regulate the rate of sampling; and (**C**) the sampling device of Foustoukos and Pérez- Rodriguez (86) that employs a series of HP and micrometering valves that facilitates gas-tight samples to be collected while minimizing cell lysis and volatile loss during decompression.

To facilitate continuous culturing, the developed systems are composed of: (i) a chemical inert pressure vessel (e.g., Ti-alloys), (ii) a gas-tight fluid delivery system, (iii) a growth medium reservoir, (iv) agitation controls, (v) temperature controls, (vi) a sampling device, and (vii) a backpressure regulator (Fig. 2). The use of Ti-alloys for the construction of the bioreactor minimizes biofouling effects (102, 103). Considering the strong impact of temperature on microbial growth and survivability, temperature control needs to be smooth and precise within 1° centigrade. Thermocouples need to be in direct contact with the culture medium inside the bioreactor, while *in-situ* agitation would minimize thermal gradients. Similarly, the backpressure-regulating valve needs to quickly adjust pressure fluctuations within a few kPa to adjust for fluid removal during sampling while maintaining a constant pressure during continuous medium delivery.

Significant technological challenges are introduced when performing gas-tight operations, sampling, and agitation under high hydrostatic pressure conditions. Earlier designs used magnetic agitators and syringe pumps or HPLC pumps for gas-tight operations (83, 96), and sampling protocols were developed to minimize cell shearing and pressure effects in the bioreactor during fluid withdrawal under HP conditions (39). The pressure ratings of the chemostat components constrain the operational limits of the HP incubation system. For example, in Foustoukos and Pérez-Rodriguez (86), even though the pressure vessel can sustain pressures up to 110 MPa, the backpressure-regulating valve and the fluid delivery pump are limited to 69 MPa. Overall, the design of these systems needs to be as simple as possible to provide the maximum degree of flexibility and even allow culturing experiments to be conducted in parallel with field studies at near *in-situ* conditions to facilitate the use of pristine microbial communities collected from extreme environments (94, 100). Flexible experimental designs that can be conducted shipboard, for example, will also eliminate the need for sample/culture transfer from the field to laboratory facilities under the physicochemical conditions of the microbial habitat (e.g., P, T, and nutrients). To address the need for a detailed description of HP continuous culturing systems, we present key protocols that can be widely applied.

### Chemostat theory

The theory of continuous culturing is well-established (e.g., references 85, 104–106). The fundamental concept is based on the continuous supply of growth medium to facilitate culturing and biomass development under steady-state conditions. In this open-system approach, microbial growth is modeled by following Monod’s equation (107):


(eq. 1) $$\mu =\mu_{max}\frac{s}{K_{s}+s}$$

where *μ* is the specific growth rate, *s* is the concentration of the growth substrate measured at the outflow of the bioreactor, and *K_s_* is the substrate concentration at which growth proceeds at half of the maximum rate (*μ*_max_). The *μ_max_* is assumed to approximate the maximum specific growth rate measured in batch mode experiments at ambient pressure (85). The *μ_max_* can also be assessed in a continuous culture when growth attains rates faster than the medium delivery rates under nutrient-rich conditions (101). The half-saturation constant *K_s_* shows the metabolic affinity of a microorganism for the specific substrate. Thus, it can constrain the relative efficiency of microbial species to dominate a mixed microbial population. It is interesting to note the scarcity of continuous culturing experiments involving mixed populations under high hydrostatic pressures (84, 94, 95, 98, 100, 108). To our knowledge, co-cultures have never been studied under HP continuous culturing conditions.

Another critical parameter in chemostat operation is the dilution rate (*D*), which is defined as the ratio between the flow rate and the volume of the bioreactor (85, 105). Under steady-state conditions for biomass growth, *D* is equal to the *μ*. Therefore, as long as the supply of nutrients is maintained, microbial cultures can be sustained for a very long time (days to months) compared to the batch culturing approaches (36, 100, 101). When *D* exceeds the *μ_max_* of the microorganisms at pressure, steady-state conditions are disturbed, and cells are washed out from the system at rates higher than the cells’ growth rates. At this point, the concentration of *s* is equal to the inflowing medium concentration (*s*_*R*_), and the critical dilution rate (*D*_*c*_) is estimated as follows:


(eq. 2) $$D_{c}=\mu_{max}\frac{s_{R}}{K_{s}+s_{R}}$$

This parameter defines the boundary conditions beyond which negative growth rates (i.e., cell washout) prevail. Thus, it provides another means to estimate the *K*_s_ by recognizing that the *μ*_max_ at conditions is equal to *D*_*c*_ and *s_R_* >>*K_s_* (85, 109). Complete wash-out of the cultured microorganisms from the chemostat occurs when dilution rates are greater than *D_c_*; a condition at which dilution occurs faster than the maximum growth rate.

### Sterilization of the experimental setup

One of the critical aspects of continuous culturing is the sterilization of the chemostat’s components. This is not often discussed in detail; however, it is a crucial step considering the size of the bioreactors and the complexity of the systems relative to the batch culture approaches. For example, sterilization by autoclaving may only be feasible for the bioreactor itself (62, 95). On the other hand, chemical sterilization can be applied to the other components (e.g., delivery pumps, pressure lines, sampling valves, and backpressure regulators) (83, 86, 96). Theoretically, culturing of extremophiles at elevated pressures (e.g., >10 MPa) prevents contamination of environmental microorganisms accustomed to the Earth’s surface conditions, especially if the growth medium is highly reducing and anaerobic as with resembling deep-sea hydrothermal vent microbial habitats (e.g., reference 110). Using pressure as a means of sterilization is also the key aspect of HP microbial treatment in the food industry (19, 21). However, HP studies of *Escherichia coli* strains have suggested that cells survive pressures way beyond 50 MPa (111–114). Furthermore, the presence of dead microorganisms in systems under extreme conditions can distort cell counting (e.g., acridine orange), and serve as a carbon source for heterotrophic growth. To this end, chemical sterilization is rendered essential even in culturing of hyperthermophilic microorganisms at elevated pressures.

For chemical sterilization, Bothun et al. (96) used exposure to absolute ethanol for 24 h. However, the low density of absolute ethanol (0.789 g/cm^3^) renders it a difficult solution to deliver. Foustoukos and Pérez-Rodriguez (86) adopted a combination of sodium hypochlorite and ethanol aqueous solutions. Subsequent HP culturing studies (36, 101) modified the specific protocol to: (i) flushing a 10% (by weight) sodium hypochlorite aqueous solution (2× volume of the reactor), (ii) followed by a 50% (vol/vol) ethanol aqueous solution for at least 6 h (>10× volume of the reactor), and (iii) rinsing and autoclaving the titanium reactor at 150°C (vapor saturation pressure) with autoclaved deionized water to remove traces of sterilizing solutions (>10× volume of the reactor). Inadequate rinsing with ethanol/H_2_O solutions (steps 2 and 3) might leave residual sodium hypochlorite that can be detrimental for the growth of the cultured community.

### Dissolved gases in growth medium

The majority of HP continuous culturing systems employ a growth medium reservoir that can accommodate the introduction of substrate gases (e.g., H_2_, CO_2_, O_2_, and CH_4_) at a range of partial pressures (e.g., 7.4 MPa CO_2(g)_ in Bothun et al. [96]). The delivery of volatile-enriched medium into the bioreactor is facilitated by (i) gas-tight syringe pumps sampling the pressurized medium solution (86, 96); (ii) HPLC systems (62, 97, 98), or (iii) direct delivery of growth medium through a piston accumulator (94). These techniques allow for the delivery of volatile-enriched medium inside the bioreactor at pressures higher than the partial pressure of the gases dissolved in the media reservoir. To avoid degassing of the dissolved volatiles upon delivery, pressure conditions in the bioreactor need to exceed those of the growth medium reservoir.

The dissolution of the volatile species is dictated by the thermodynamic equilibrium between the gas and liquid phase, expressed by the Henry’s law constant described as:


(eq. 3) $$K_{H}=\frac{M_{g}}{p_{g}}$$

where *M*_g_ is the molarity (mol/L) and *p*_g_ is the partial pressure (atm) of gas in equilibrium with the aqueous solution (e.g., reference 115). The equilibrium constant of this reaction depends on pressure and temperature. It can be calculated based on the thermodynamic properties of the aqueous and gas species attained from well-established thermodynamic databases (e.g., http://geopig3.la.asu.edu:8080/GEOPIG/pigopt1.html, 116).

**Fig 3:**
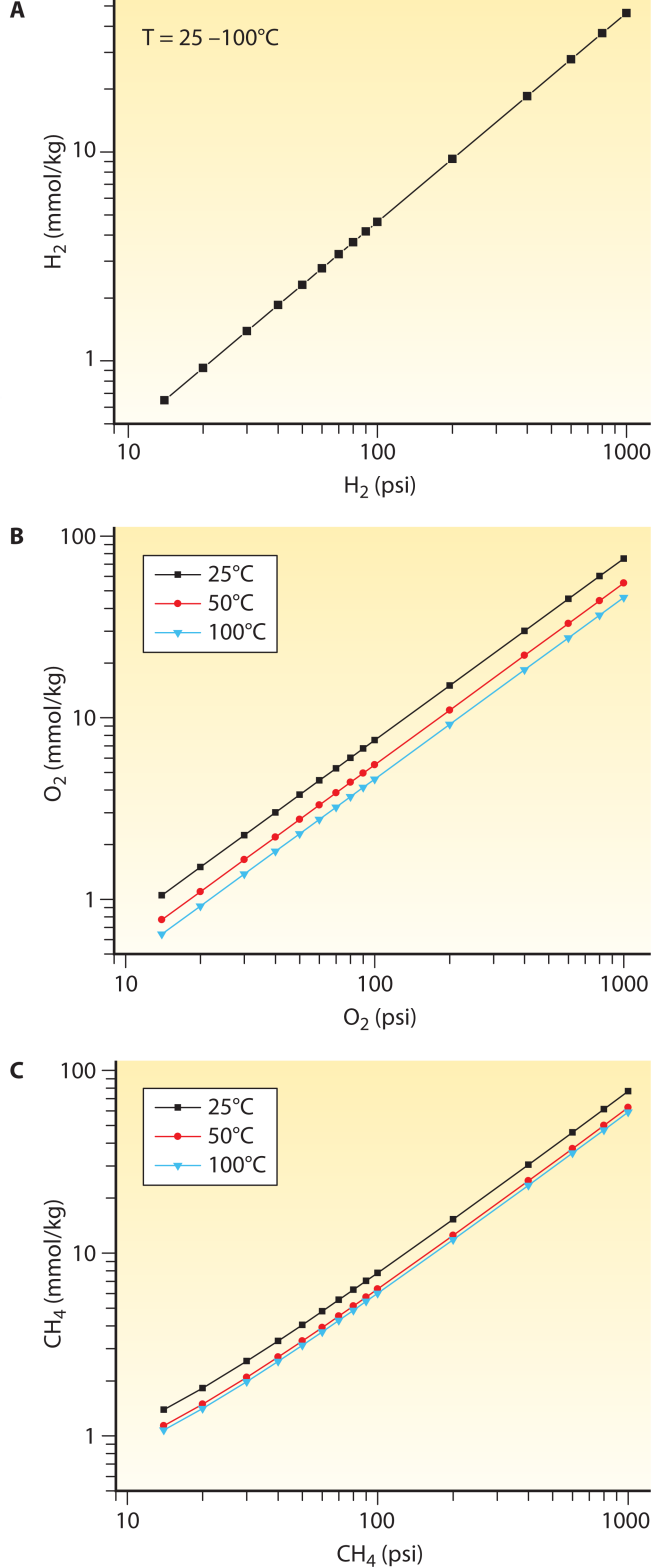
The solubility of H_2_ (**A**), O_2_ (**B**), and CH_4_ (**C**) as a function of temperature and the partial pressure of gases in equilibrium with a 3.2 wt% NaCl aqueous solution. The effect of the ionic strength of the solution on gas solubility is based on theoretical and empirical models developed by Drummond (117), Tromans (118), and Duan et al. (119) for the H_2_, O_2_, and CH_4_, respectively. This effect is not very prominent for relatively dilute NaCl solutions; thus, the concentrations of dissolved gases in a pure H_2_O can also be used for a first approximation.

In Fig. 3, the solubility of H_2_, O_2_, and CH_4_ is shown as a function of the partial pressure of the gas (in the growth medium reservoir) and temperature. The speciation model applies for a 3.2 wt% NaCl-H_2_O solution (i.e., seawater salinity) (117–119) with fitted parameters presented in Table 1. With O_2_ and CH_4_, for example, increasing temperature results in decreasing solubility. This might affect O_2_ delivery from a growth medium reservoir equilibrated at room temperature to cultures growing at hyperthermophilic or thermophilic conditions. This is not the case for the solubility of H_2_, which shows very little dependence on temperature up to 100°C. To attain dissolved H_2_, O_2_, and CH_4_ concentrations commonly found in seafloor hydrothermal or seep environments (e.g., >10 mM), the medium reservoir needs to sustain pressures higher than 200 psi (1 psi = 6.895 kPa).

**TABLE 1:** Fitted parameters to describe the solubility of H_2_, O_2_, and CH_4_ as a function of temperature and the partial pressure of gases (10–1,000 psi) in equilibrium with a 3.2 wt% NaCl aqueous solution*^a^*^,^*^b^*

Gas	*T* (°C)	*a*	*b*
H_2_	25–100	0.0462	null
O_2_	25	0.0750	0.140
	50	0.0551	null
	100	0.0459	null
CH_4_	25	0.0761	0.423
	50	0.0622	0.165
	100	0.0588	0.173

^*a*^ The linear regression model adopted is in the form of (mmol/kg) = a*(psi) + *b* as shown in Fig. 3.

^*b*^ All the linear regression models exhibit *r*^2^ > 0.9999 and *P* value < 0.005.

**Fig 4:**
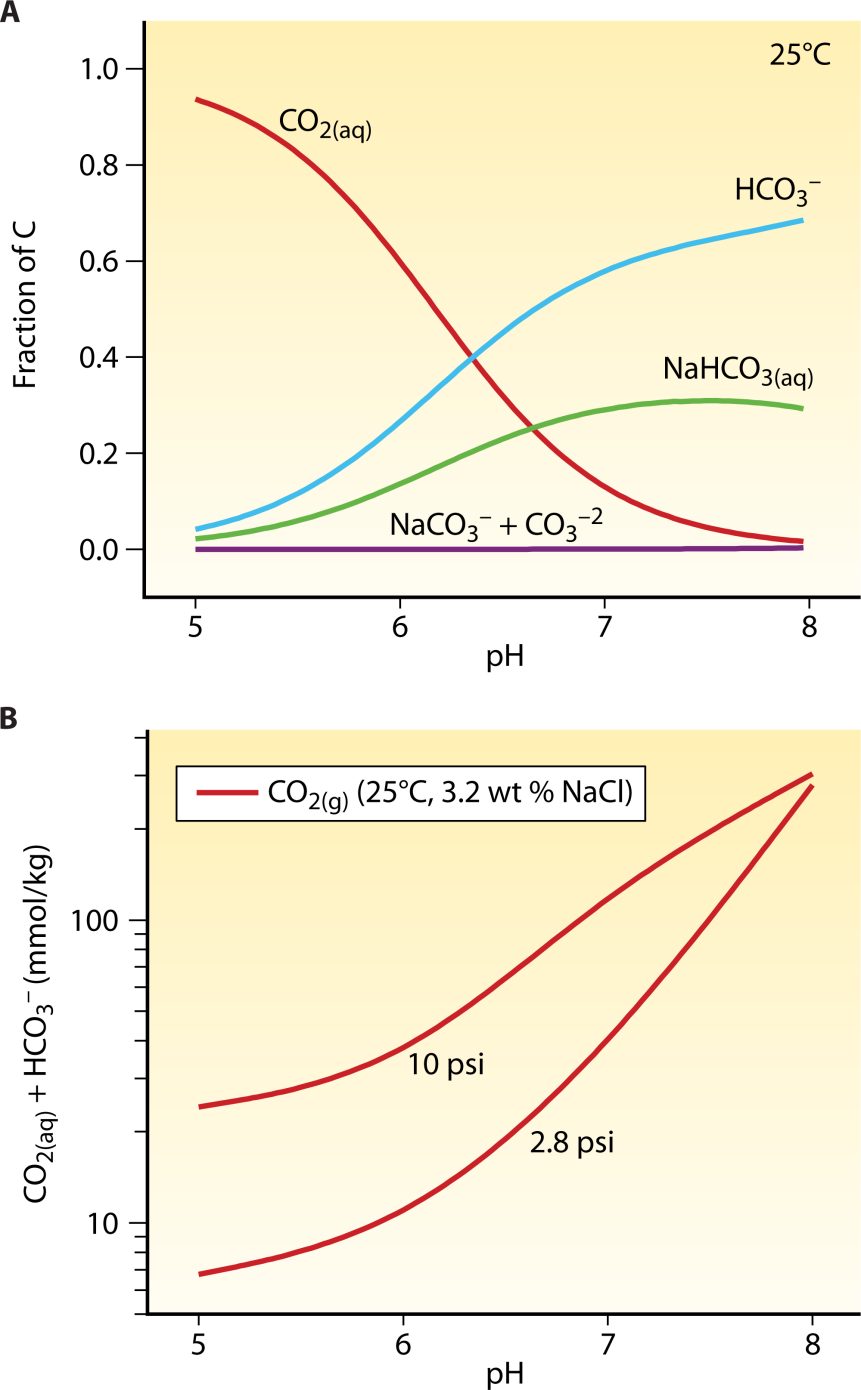
(**A**) A speciation model for a 3.2 wt% NaCl aqueous solution in an equilibrium with H_2_/CO_2_ (80:20) mixture at 14 psi (i.e., 96.5 kPa) partial pressure. (**B**) The concentrations of the bioavailable dissolved CO_2_ (CO_2(aq)_ + HCO_3_^−^) depend on the CO_2(g)_ partial pressure, the pH, and the NaCl content of the aqueous solution.

In the case of CO_2_, gas solubility depends on temperature, the partial pressure of CO_2(g)_, fluid salinity, and pH (Fig. 4) [e.g., (115)]. Speciation models need to account for Na-bearing species, such as NaHCO_3(aq)_ and NaCO_3_^-^ (120). Acidic conditions favor CO_2(aq)_ (i.e., H_2_CO_3(aq)_), while HCO_3_^−^ and NaHCO_3(aq)_ are the dominant species at near-neutral pH conditions (Fig. 4A). At pH ~6.5 (25°C), there are equal concentrations of CO_2(aq)_ and HCO_3_^−^ accompanied by elevated NaHCO_3(aq)_ concentrations (Fig. 4A). Carbon bioavailability in autotrophic metabolism is likely determined by the relative abundances of CO_2(aq)_ and HCO_3_^−^ rather than of the Na-bearing species (121, 122) (Fig. 4B). Thermodynamic calculations conducted for a 3.2 wt% NaCl-H_2_O solution indicate that for 80:20 mixtures of H_2_/CO_2_ gases at a partial pressure of 14 psi (i.e., 2.8 psi CO_2(g)_ in the medium reservoir), the total concentrations of dissolved CO_2(aq)_ and HCO_3_^−^ can vary from 7 to 280 mmol/kg at pH 5 and 8, respectively (Fig. 4A).

### Inoculation protocols

Inoculation protocols generally involve the growth of a preinoculum community inside the bioreactor as a batch culture before initiating continuous medium flow (83, 86, 96, 104). Before this stage, control experiments at pressure and temperature can be performed to evaluate the level of environmental contamination in the experimental setup and the growth medium reservoir. The inoculation protocol developed in recent studies (36, 86, 101) follows two stages. In the initial stage, an abiotic control experiment is conducted by prefilling the bioreactor with a sterile growth medium and allowing over 24 h of pre-treatment at the pressure and temperature conditions of the continuous culturing experiment. Samples collected during this period assess contamination by living or dead environmental microorganisms by direct cell count and by monitoring the composition of growth substrates (e.g., H_2_, O_2_, HCO_3_^−^, and yeast extract) at the outflow to verify a lack of substrate consumption. The second stage involves introducing a highly dense microbial population (preinoculum) in the bioreactor. In general, a volumetric ratio of 30–50 between preinoculum and bioreactor is adopted; however, inoculations may also be commenced at significantly lower ratios [e.g., ratio = 3 (99)]. After bioreactor inoculation, the system remains in batch or under a very low fluid flow rate continuous culture (e.g., <1/2,000 bioreactor volume [mL]/min) mode and under the pressure conditions of the growth medium reservoir until the microbial community attains cell densities comparable to those of the initial preinoculum. If the levels of nutrient substrates have been maintained at levels non-limiting for growth, then the rates of microbial growth attained at this stage may correspond to the maximum growth rates of the microorganisms at the imposed pressure/temperature conditions.

### Mixing and agitation protocols

HP continuous culturing systems need to allow for the homogenous mixing of delivered growth medium with the cultures in the bioreactor. Techniques employed are a magnetic stirrer (83) or a shaft-driven magnetic agitator (96) (e.g., Fig. 2A). The latter introduces challenges associated with the need for a HP (and temperature) sealing surface between the internal agitator and the external magnetic driver. In both approaches, mixing at high pressures (>5 MPa) requires drivers strong enough to overcome internal stresses on the stirring mechanism. Recent technological advances permit magnetic agitation at speeds as high as 500 rpm at 50 MPa (86).

Not much attention has been given to the protocols for the internal mixing of cultures and medium to address the physicochemical conditions of the different operational stages, such as sterilization, initial growth medium introduction, culturing, and sampling. Agitation is permitted during sterilization and culturing to facilitate the homogeneous mixing and distribution of fluids and cultures in the bioreactor. However, it needs to be restrained during the initial introduction of growth medium and sample collection to allow for the efficient medium transfer and retrieve samples representative of the cultured community. Details on the effect of agitation on sampling along with the associated operational protocol are discussed in “Sampling protocols,” below.

**Fig 5:**
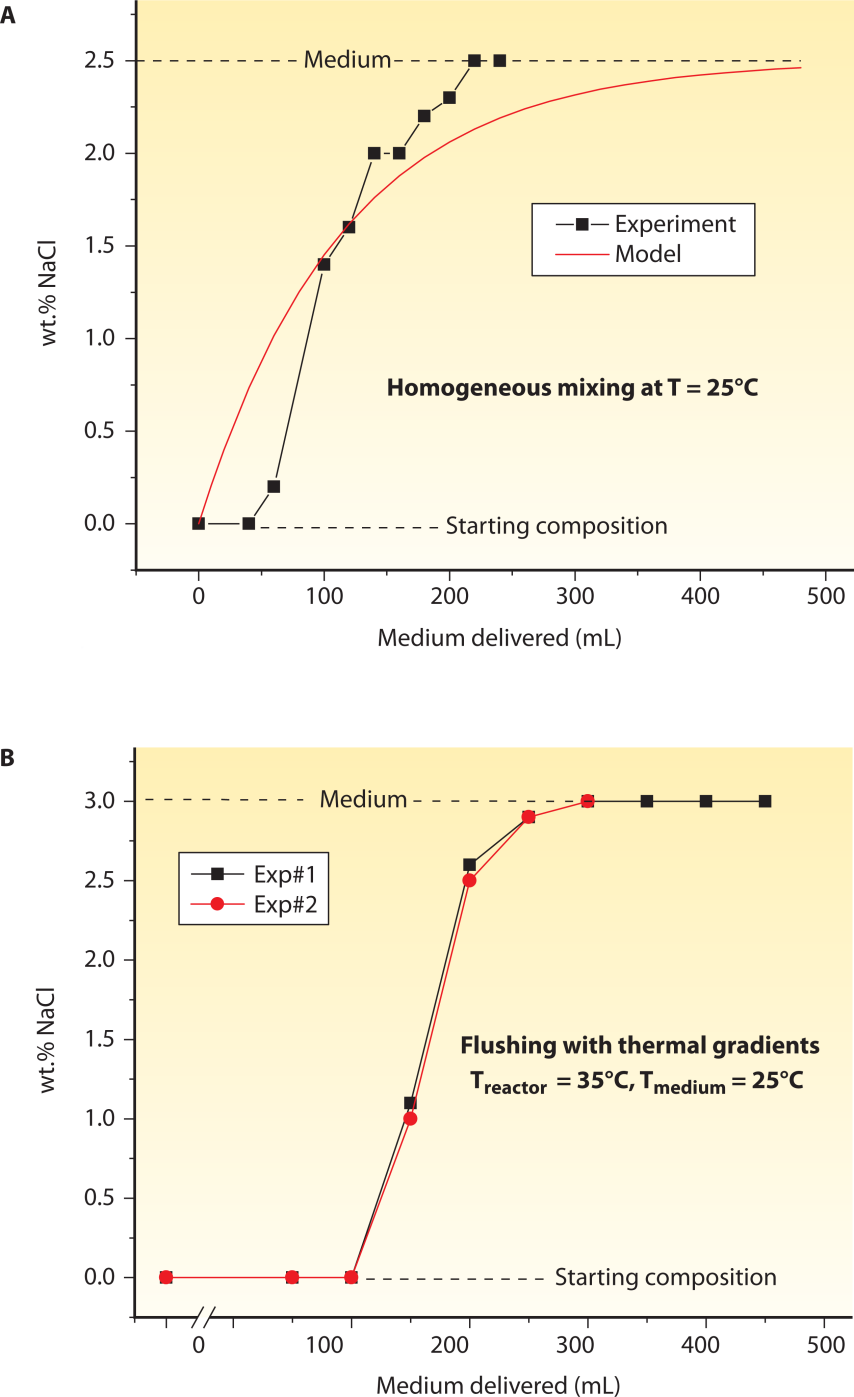
The evolution of medium introduction in the bioreactor during the expulsion of effluent H_2_O. These experiments have been conducted in a 110 mL volume reactor (86). (**A**) In the homogenization experiments, the composition of the mixture inside the reactor depends on the rates of fluid flow and the composition of the influent solution. Data are in close agreement with theoretical models on chemical reactor fluid mechanics (123). (**B**) By developing strong thermal and density gradients between the influent and effluent, the expulsion rate of the latter is significantly higher than under homogeneous mixing (**A**).

After the initial stages of bioreactor sterilization (see “Sterilization of the experimental setup,” above) and before the inoculation protocols (see “Inoculation protocols,” above), the bioreactor is prefilled with growth medium by removing fluids involved in chemical sterilization (e.g., H_2_O and ethanol/H_2_O). The volume of the medium required in this stage depends on the effluent and influent solutions' thermal/density conditions and the extent of internal homogenization during fluid transfer. To evaluate the effect of thermal and density gradients and agitation on the rates of influent transfer in the bioreactor (110 mL volume), we conducted a series of ambient pressure experiments involving H_2_O (effluent), which is the final solution in the sterilization procedures, and a NaCl-H_2_O solution (influent) that represents growth medium with seawater-like salinity (i.e., 3.2 wt% NaCl) (Fig. 5). In the homogenization experiment (Fig. 5A), agitation was maintained at 110–130 rpm. In the direct flushing experiments (Fig. 5B), the effluent was maintained at 35°C, while the saline and dense influent were at ambient temperature conditions (Fig. 5B). Results indicate that the volume requirement for a complete influent transfer is nearly two times greater (~2 times bioreactor’s volume) during homogenization than by flushing under thermal and density gradients. This has important implications when the volume of growth medium is limited, such as when it involves influent fluids collected directly from natural microbial habitats [e.g., hydrothermal vent fluids, (67, 100)].

Furthermore, the homogenization rates are expected to play a key role in experiments focusing on the microbial response and adaptation to varying nutrient concentrations. For example, when transitioning between different growth media, the volume of influent required to replenish the media inside the reactor fully should be more than twice the bioreactor’s volume, assuming conditions of continuous agitation. These volume constraints, along with the imposed dilution rates, would control the time needed to establish a new steady state for the microorganisms cultured under constant pressure and temperature conditions (101).

To this end, our established protocol for growth medium transfer requires (i) effluent H_2_O to be at a higher temperature than the influent medium, and (ii) medium to be delivered at relatively modest flow rates (<2 mL/min) in the absence of agitation to minimize disturbing the thermal and density gradients between the effluent and influent solutions.

### Sampling protocols

A crucial step of HP culturing is collecting representative samples from the communities and the growth medium by maintaining the integrity of cell structures and minimizing volatile loss during decompression. Early experimental developments demonstrated the impact of sampling protocols on the bioreactor’s pressure conditions and the integrity of the microbial cell structure collected (38, 39). These studies revealed that sampling not only may induce decompression effects in the bioreactor but also result in cell lysis because of the large shear forces imposed by the decompression of collected biomass through narrow tubing and HP valve stems. The former is not crucial for continuous culturing experiments because a backpressure regulator controls pressure conditions under continuous fluid flow conditions. However, the retrieval of large volume samples to accommodate chemical and molecular (e.g., proteomics and metatranscriptomics) might be challenging for maintaining constant pressure conditions even for chemostats.

The earlier designs of Yayanos (38) and Taylor and Jannasch (39) involved using a series of valves and sampling tubes that allowed the collection of samples along minimal pressure gradients between bioreactor and sampling device. Samples are collected by allowing for undisturbed fluid flow through a wide-open HP valve directly attached to the bioreactor. At the other end of the subsampling devices, HP valves and pumps regulate the rate of sample retrieval. Constant pressure conditions are maintained by introducing a moving piston in the subsampling chamber that separates the introduced sample from the pressure transmitting medium (i.e., H_2_O) and meters the sampling rate (39) (Fig. 2B).

Elements of these designs have been implemented in HP culturing methods. For example, the protocol of Sauer et al. (62) isolates samples in pre-conditioned (e.g., vacuum) sampling tubes, while Bothun et al. (96) used a small sampling loop to minimize decompression effects. A similar approach appears to be adopted in Parkes et al. (124). Recent developments implemented the approach of Taylor and Jannasch (39) to permit sample retrieval without affecting the pressure of the microbial community inside the bioreactor and without subjecting microbial cells to high shear forces (67, 86).

**Fig 6:**
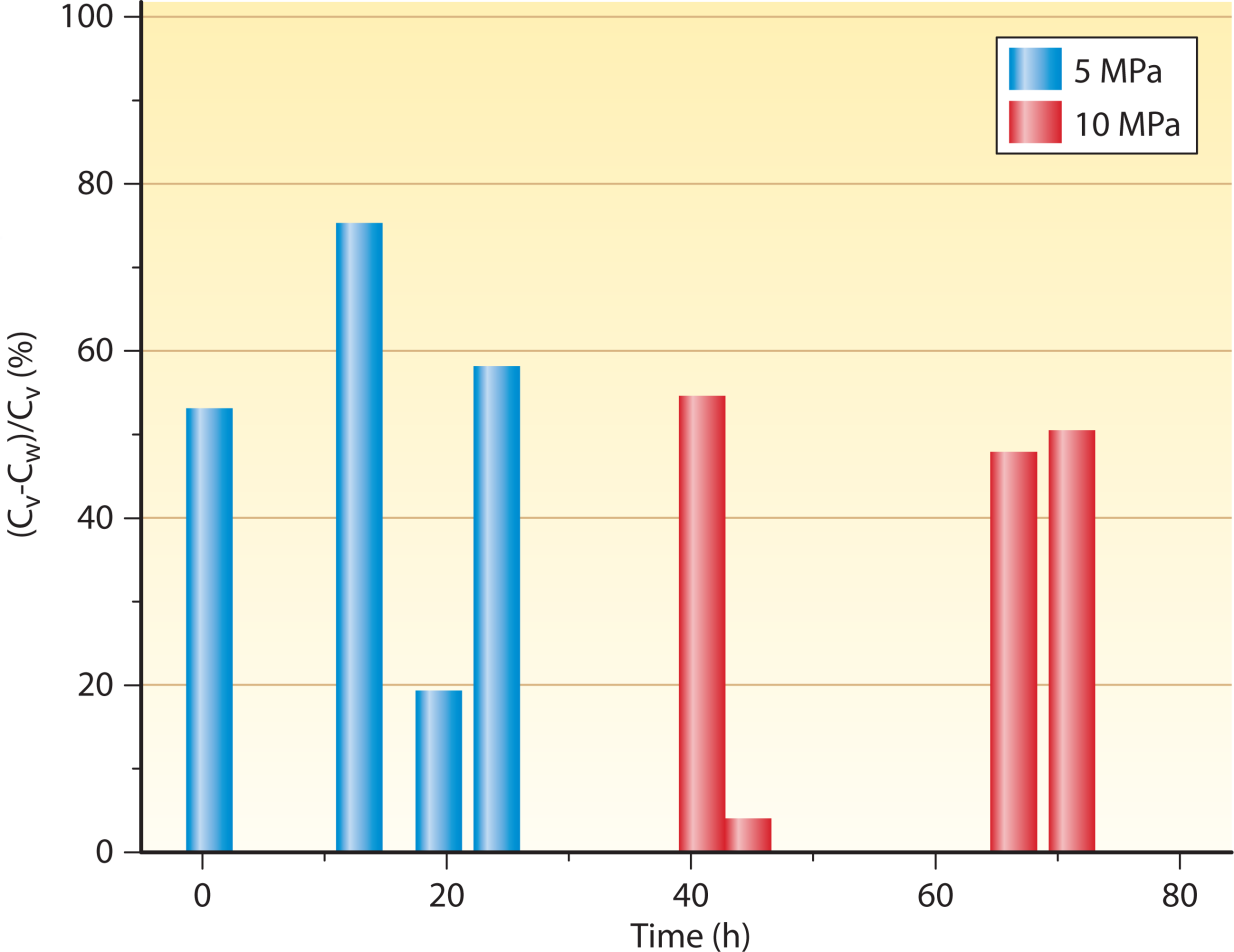
The deviation in cell densities attained between samples collected from the outflow (C_w_) and retrieved by following HP sampling protocols (C_v_) (86). The deviation is determined as the percent ratio of (Cv-Cw)/Cv for the HP continuous cultures of *Thiomicrospira thermophila* conducted at 5 (<25 h) and 10 MPa [unpublished data, (101)]. Sampling methods lacking adequate HP protocols are deficient, as indicated by the approximately 50% average (Cv-Cw)/Cv ratio and its considerable variation around 40 h (10 MPa).

Even though the effect of sampling on cell lysis has been suggested to be prominent, the experimental evidence is very limited. Park and Clark (97) investigated the effect of decompression rate on the density of the HP culture of *Methanococcus jannaschii*. Results showed substantial cell lysis when samples were collected in 1 s relative to slower sampling rates (5 min). Similarly, a strong negative impact on the density of collected biomass has been linked to the shear forces developed when forcing cells through the narrow stems of HP valves (125, 126). Samples collected directly from the outflow of HP continuous culture of *Thiomicrospira thermophila* indicate a nearly 50% depletion in cell density relative to samples collected by employing protocols that minimize cell lysis during fluid retrieval from HP cultures (Fig. 6) (101).

Few studies present the adopted sampling protocols in detail (38, 39, 67, 86). For example, in Foustoukos and Pérez-Rodriguez (86), subsampling is performed by operating three successive HP valves. Samples are collected within the dead space of the valves and the titanium tubing that connects the valves (Fig. 2C). The assembly is prefilled with autoclaved deionized water to minimize depressurization in the sampling device when opening the valve to the bioreactor. Upon fully opening the valve directly connected to the chemostat and the intermediate valve, sampling commences by adjusting the position of the outlet micrometering valve to maintain the HP conditions inside the bioreactor. At this point, agitation is stopped to minimize influent contributions to sampling fluids, and delivery of growth medium is increased to minimize the drop in pressure [e.g., <1% pressure fluctuation at 50 MPa, (83)]. Through the outlet micrometering valve, fluid samples are collected in gas-tight syringes for chemical analysis. Upon completion of this stage, sampling stops and the continuous culturing resumes. The sampling arrangement is isolated from the bioreactor and the cells trapped inside the medium-filled dead space of the sampling titanium tubing and valves (Fig. 2C) represent the intact microbial population cultured in the bioreactor. This protocol minimizes cell lysis effects imposed by the decompression of the HP culture and fluid flow through the stem of the outlet micrometering valve while maintaining the integrity of chemical analyses, particularly of dissolved gases. A similar approach was employed by Mori et al. (68) and Oliver et al. (69), with the latter introducing novel techniques for HP filtration without decompression.

If the volume of samples collected is significantly smaller than the bioreactor’s volume (e.g., <10%), then the influent media has a small contribution to the overall dilution rates and imposes minimal effects on the extent of media-culture homogenization (see “Mixing and agitation protocols,” above). Nevertheless, the estimated net dilution rates need to account for the influent’s pulses introduced during sampling (101).

## FUTURE DIRECTIONS

The protocols described for HP continuous culturing have been developed from monoculture-based studies. However, extremophiles grow as communities where they interact and exchange genetic material and metabolites (e.g., 110, 127). In deep-sea vent ecosystems associated with ultramafic-hosted hydrothermal systems (e.g., 128), for example, autotrophic H_2_-fueled microorganisms supply food and energy needs for coexisting heterotrophic communities, which in turn, produce H_2_ through fermentation or carboxydotrophy (129–131). Despite the importance of autotrophic-heterotrophic symbiosis for the habitability of extreme environments, the limited studies conducted have explored the syntrophic growth of hyperthermophilic archaea (132, 133) and the cycling of H_2_ within microbial biofilms composed of heterotrophic and H_2_-utilizing autotrophic bacteria (134). There is a lack of syntrophy studies involving extremophilic microorganisms at high-pressure conditions (7) .

In the extreme abyssal environments of hydrothermal vents, an exchange can also occur between microbes and viruses. Earlier studies have shown that in low-temperature diffuse flow vent fluids, viral abundances are significantly higher than those encountered in the deep ocean water column (135, 136). Vent fluids are also enriched in lysogenic bacteria that contain prophages (136–138). With a few bacterial and archaeal phages isolated and sequenced (139), the ecological and evolutionary impact of viral populations at hydrothermal vents is unconstrained. Future HP continuous culturing experiments of prophages-host bacteria and syntrophic microorganisms are expected to introduce additional elements in the culturing protocols, especially for the inoculation and sampling procedures.

Future HP culturing designs may also adopt technological advances related to the *in-situ* characterization and monitoring of aqueous solutions. For example, advances in electrochemistry have introduced robust methodologies to develop efficient and straightforward electrochemical cells to measure dissolved H_2_, H_2_S, and pH at a range of temperature, pressure, and chemical conditions (140–145). These are solid-state electrodes constructed of metal/metal oxides (e.g., Au, Pt, Ir/IrO_2_, ZrO_2_ [9% Y_2_O_3_], Ag/Ag_2_S, and Ag/AgCl), exhibiting resistance to corrosion and able to respond under elevated temperatures and pressure conditions electrochemically. Electrode-based chemical sensors (e.g., Au/Hg) have also been developed to analyze for dissolved O_2_, Fe^+2^, Mn^+^, and HS^−^ (146), while recent developments have shown the efficiency of solid-contact polymeric ion-selective (K^+^ and Na^+^) electrodes for high-pressure (10 MPa) applications (147). These electrode-based sensors can be placed in contact with the incubated growth medium through high-pressure/-temperature coupling connections. The small surface area and the relatively inert nature of the electrodes are expected to impose minimal effects on the physicochemical conditions of HP culturing experiments. Future studies, however, need to evaluate the extent of biofouling on the metal/metal oxide surfaces of the electrochemical cells. Finally, Raman vibrational spectroscopic methods involving the employment of miniature fiber optic probes in contact with cultures and growth media may significantly expand the range of chemical species (CO_2_, CH_4,_ and SO_4_^2−^) monitored *in-situ* at the challenging conditions of HP continuous culturing studies (148–151).

With no doubt, advances in HP continuous culturing will accelerate studies involving petroleum biodegradation, biofuel synthesis *ex-situ*, and in subsurface reservoirs, food processing, and the evolution of biogeochemical cycles at pressures and temperatures resembling those of extreme planetary biospheres.
